# Beaver Tail Liver: A Hepatic Morphology Variant

**DOI:** 10.7759/cureus.16327

**Published:** 2021-07-12

**Authors:** Sachin Khanduri, Saif Malik, Nazia Khan, Harshika Singh, Mufidur Rehman

**Affiliations:** 1 Radiology, Era's Lucknow Medical College and Hospital, Lucknow, IND; 2 Radiodiagnosis, Era's Lucknow Medical College and Hospital, Lucknow, IND

**Keywords:** liver, morphology, anatomic variation, sliver of liver, beaver tail

## Abstract

Beaver tail liver, or else known as the sliver of liver, is a rare anatomic variation of the liver where the left lobe of the liver extends laterally to contact and enwrap the spleen.

A case is presented here where a middle-aged male presented with complaints of abdominal pain, hematuria, and fever. After the routine blood and urine examinations revealed urinary tract infection, CT abdomen was done to find out the etiology, and beaver tail liver was found incidentally with the left lobe of liver encircling the spleen.

Sometimes it may be difficult to differentiate liver and spleen from each other when echogenicity or density on USG and CT are equivalent. More common in females, it may imitate a splenic trauma or a subcapsular hematoma, or a perisplenic hemorrhage within the splenic parenchyma.

## Introduction

Beaver tail liver, also called sliver of liver, is a rare variant of liver morphology. Sometimes elongated left lobe of the liver can extend laterally across the midline to contact and often surround the spleen [1-2]. They are predominantly found in females and generally but not always detected as an incidental finding in the patient. It is more prone to injury in case of trauma occurring to the lower left chest or in the upper quadrant of the left side of the abdomen and it can be misdiagnosed as a subcapsular hematoma of spleen or perisplenic fluid collections. The liver and spleen show similar echogenicity on USG and density in CT especially in cases of abdominal trauma as the two organs are not easily differentiated [1-2].

## Case presentation

**Figure 1:**
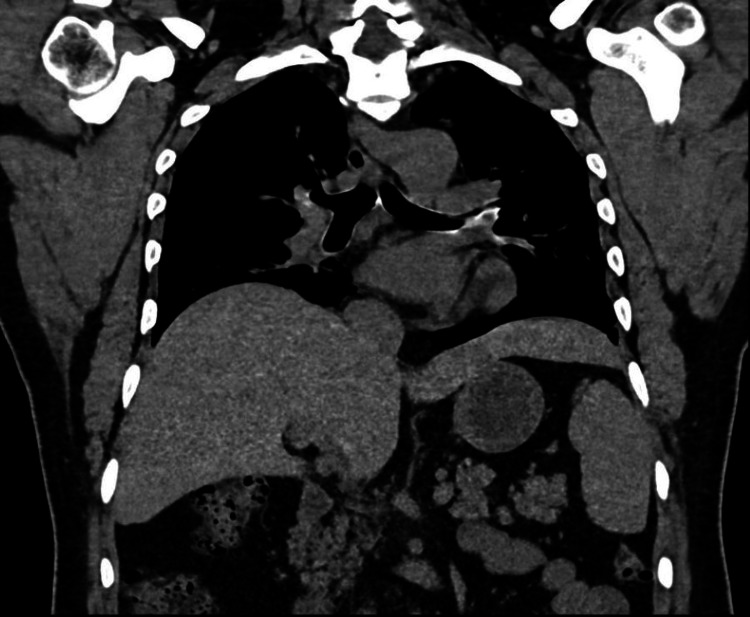
CT demonstrating beaver tail liver.

**Figure 2:**
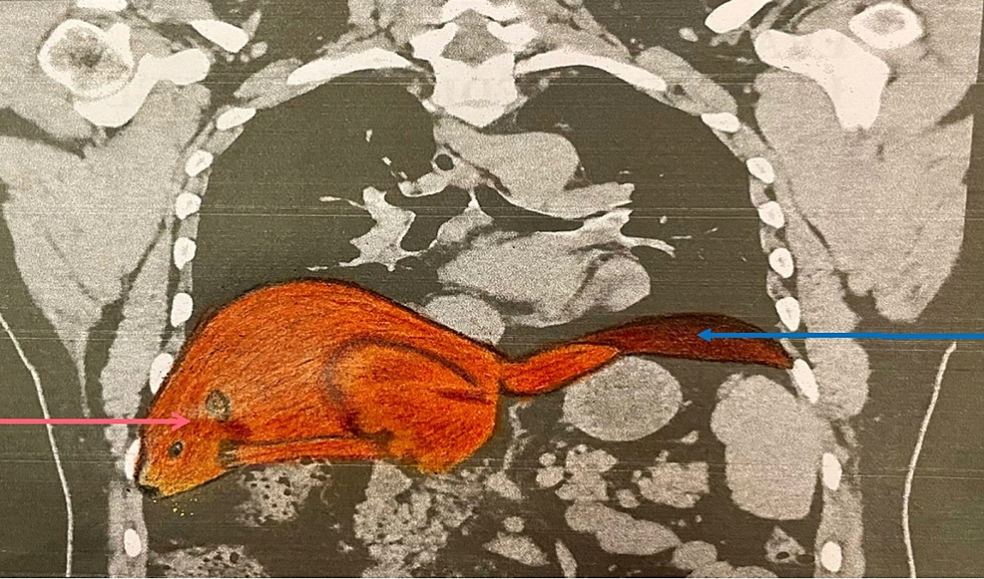
CT demonstrating elongated left lobe of liver mimicking a beaver's tail (blue arrow) with normal right lobe of liver (red arrow).

A 42-year-old man presented to the Medicine Outpatient Department (OPD) of Era’s Lucknow Medical College and Hospital, Lucknow with complaints of abdominal pain (in the left upper quadrant radiating to front), fever, and hematuria. On general physical examination, his vitals were found to be stable with heart rate and blood pressure within normal range. Blood investigations revealed an increase in the leucocyte count with neutrophilia. In routine urine examination, WBCs, RBCs, and pus cells appeared. In urine culture, the organism was found to be Escherichia coli (E. coli). Following which the appropriate treatment was given but subsequently USG and CT abdomen were done to find out the etiology and rule out the obstructive uropathy as he had similar episodes in the past also which demonstrated renal calculi in the calyx of bilateral kidneys. Incidentally encountered, his liver was found to be exceptionally elongated along with splenomegaly, in which the left lobe of the liver was extending up to the spleen and nearly encircling it. This anatomic variation of the liver is known as beaver tail liver (Figure 1) as it resembles the tail of a beaver (Figure 2).

## Discussion

Lobar anatomic variants of the liver are not very commonly found. There can be ectopic liver lobes (which are found separately from the liver with no anatomical continuation) and accessory lobes of the liver (which are extra lobes of the liver having anatomical continuity with the main organ in the form of normal liver parenchyma and having biliary ducts and hepatic vessels) [3]. Accessory lobes are more commonly found in the right lobe of the liver, generally beneath the liver [3]. They can have a fixed or pedunculated form of attachment and are diagnosed by imaging as an incidental finding or if torsion develops, especially of the pedunculated form [3]. One of the most commonly found accessory lobes corresponds to hypertrophied segment V and VI best known as Riedel’s lobe [3].

The left lobe may also vary from its normal size and shape, although the hepatic parenchyma has normal echotexture. While they pose no additional risk for hepatic pathologies due to this, they can sometimes have clinical implications [4]. Occasionally, an anatomical variant of the liver known as “beaver tail liver” can be found incidentally. It is more commonly found in females. There can be two commonly occurring conditions for a beaver tail liver. One involves the lateral segment of the left lobe of the liver extending laterally across the midline exceeding the anterior border of the spleen and almost or virtually encircling it [4].

The focused assessment with sonography in trauma (FAST) exam has become an important screening tool to assess the patient of blunt abdominal trauma by looking for the presence or absence of hemoperitoneum. This is important since the initial management of such patients depends on detection of hemoperitoneum but a difference in echotexture of liver and spleen. This extended lobe of the liver can be misdiagnosed as subcapsular hematoma of spleen or perisplenic fluid collection and if the radiologist is not aware of normal variant, it could lead to a false-positive diagnosis [5]. Also, the blunt injuries to the left upper quadrant, which normally affects the spleen, may involve this elongated left lobe of the liver which can again be mistaken for splenic hematoma [6]. If the echogenicity of liver parenchyma is similar to the spleen, it can be mistaken as a splenic mass on USG and CT scans [4].

The perisplenic region should be evaluated carefully so as to ascertain whether hepatic and portal veins are present or not. Color Doppler can also be done to identify the normal hepatic and portal vessels [1]. If a perisplenic density is found especially along the superior and lateral aspect but does not have any evidence of bleeding in the peritoneal region like irregular margins of the spleen, fracture planes passing through the spleen, hypo or hyperdense spleen, and no significant clinical findings, then one should strongly consider that the left lobe of the liver is encircling around the spleen instead of perisplenic hematoma [7]. This variation can also prove to be fatal if invasive abdominal procedures are done unknowingly [1].

The other condition can be a fibrous appendix of the liver which is a fibrous connective-tissue (atrophied hepatic tissue) process that extends from the lateral end of the left lobe of the liver and passes with the left triangular ligament to get attached to the diaphragm [8]. It may have various shapes but regardless contain liver parenchyma along with the blood vessels (like a hepatic artery, portal vein, hepatic vein) and bile ducts [8], thus it is an important structure and ligation should be done with care [8]. Rarely the liver tissue in the left triangular ligament can show hepatic pathologies.

## Conclusions

Beaver tail liver is a rare anatomical variation where the left lobe of the liver extends across the midline to contact and sometimes encircle the spleen. It can be involved in cases of blunt abdominal trauma which normally involves the spleen and can be misdiagnosed as perisplenic hemorrhage/subcapsular hematoma or splenic mass depending on whether it has the same or different echotexture to spleen, leading to false-positive diagnosis. It can be diagnosed as liver tissue by evaluating the normal hepatic and portal vessels with the help of color Doppler flow.
